# 酚类化合物混合暴露与男性精液质量的关联分析

**DOI:** 10.3724/SP.J.1123.2023.09009

**Published:** 2024-02-08

**Authors:** Xiaoli SHEN, Weifeng TANG, Junxia LIU, Junjie AO, Xiaoning LIU, Xian HUANG, Jin QIU, Jun ZHANG, Qianlong ZHANG

**Affiliations:** 1.上海交通大学医学院附属新华医院, 环境与儿童健康教育部和上海市重点实验室, 上海 200092; 1. Ministry of Education-Shanghai Key Laboratory of Children’s Environmental Health, Xinhua Hospital,Shanghai Jiao Tong University School of Medicine, Shanghai 200092, China; 2.上海交通大学医学院公共卫生学院, 上海 200025; 2. School of Public Health,Shanghai Jiao Tong University School of Medicine, Shanghai 200025, China; 3.上海交通大学医学院附属仁济医院, 上海 200135; 3. Renji Hospital,Shanghai Jiao Tong University School of Medicine, Shanghai 200135, China

**Keywords:** 高效液相色谱-串联质谱法, 酚类, 精液质量, 混合暴露, 男性生殖健康, high performance liquid chromatography-tandem mass spectrometry (HPLC-MS/MS), phenols, semen quality, mixed exposure, male reproductive health

## Abstract

酚类化合物是一类常见的环境内分泌干扰物,流行病学研究表明酚类化合物可能影响男性精液质量,但结果迥异。此外,现有的研究大多局限于单一酚类化合物的影响,忽视了现实情况中多种酚类物质混合暴露的健康影响,酚类化合物混合暴露与男性精液质量的关联仍需进一步探讨。本研究将所建立的高效液相色谱-串联质谱(HPLC-MS/MS)方法应用于大样本人群(799名向上海人类精子库捐赠精子样本的志愿者)尿样中18种酚类化合物的浓度测定,并探索了酚类暴露与精液质量之间的关联。通过测量精液指标(包含精子浓度、精子总数、精液体积和精子前向运动百分比),采用多元线性回归和加权分位数之和模型(WQS)分别探索单一酚类化合物暴露和多污染物混合暴露与精液质量参数间的关联。调整潜在的协变量后,多元线性回归模型发现尿液中对羟基苯甲酸乙酯(EtP)暴露与男性精子浓度和精子总数呈显著负相关(*P*<0.05);WQS混合暴露模型发现,酚类化合物混合暴露与精子浓度下降相关,其中对羟基苯甲酸甲酯与EtP是起主要影响作用的化合物。综上,酚类暴露可能与男性精液质量下降相关,主要表现在降低精子浓度和精子总数。

## Abstract (en)

Phenols such as bisphenols, parabens, and triclosan are common environmental endocrine disruptors. Previous epidemiological studies have suggested that phenols may affect semen quality, but the results were inconsistent. In addition, most existing studies have been limited to the effects of a single chemical compound, ignoring the health effects of mixed exposure to multiple chemicals. Thus, we aimed to explore the associations between individual and mixed exposure to phenols and various semen quality parameters.

In this study, a rapid and sensitive method was used to determine 18 phenolic compounds in urine samples of 799 volunteers who donated sperm samples to the Shanghai Human Sperm Bank. A spot urine sample was collected from each subject on the day of their clinic visit and stored at -20 ℃ until testing. Urine samples (200 μL) were extracted and added with 20 μL of an internal standard and 50 μL of *β*-glucuronidase solution. The mixtures were then incubated for 12 h at 37 ℃. After hydrolysis, the samples were extracted twice using ethyl acetate (500 μL). The concentrations of the 18 phenolic compounds were measured using high performance liquid chromatography-tandem mass spectrometry (HPLC-MS/MS). Semen quality parameters were analyzed using a computer-aided semen analyzer. Multiple linear regressions were used to detect the associations between individual phenol exposure and semen quality parameters. In addition, weighted quantile sum (WQS) models were used to explore the associations between mixed-phenol exposure and semen quality parameters. After adjusting for potential covariates, the results of multiple linear regressions showed that exposure to ethyl paraben (EtP) was significantly negatively associated with sperm concentration and total sperm count (*P*<0.05). In addition, exposure to mixed phenols was significantly associated with decreased sperm concentration; methyl paraben (MeP) and EtP were identified as the main contributors to this decrease. Thus, phenol exposure may be associated with decreased semen quality in young males, particularly with respect to sperm concentration and total sperm count.

不孕不育的患病率逐年上升,全球约有8%~12%的夫妇存在不孕不育的困扰,而其中约50%可归因于男性相关的因素^[1]^。影响男性生殖健康的因素十分复杂,如:饮食结构^[2]^、不良生活习惯(如熬夜、酗酒、长期吸烟)^[3⇓-5]^、特殊职业(如高温、化学物质、辐射)^[6,7]^等。此外,环境内分泌干扰物(endocrine disrupting chemicals, EDCs)暴露对男性生殖健康的影响也日益受到人们的关注。酚类化合物是一类常见的环境内分泌干扰物,包括双酚类(bisphenols)、对羟基苯甲酸酯类(parabens)、羟基苯甲酮类(hydroxybenzophenones)和抗菌剂等。酚类化合物广泛应用于工业生产、生活消费品、食品包装等^[8,9]^。由于其暴露广泛,在日常生活中人们不可避免地通过呼吸、饮食和皮肤等途径接触酚类化合物^[10,11]^。

近年来,越来越多的流行病学研究报道了酚类化合物与男性精液质量间的关联。一项横断面研究从不育门诊纳入了190名男性,测量了其尿液中的双酚A(bisphenol A, BPA)浓度,结果提示BPA浓度与精子浓度、形态异常和精子活力呈显著负相关^[12]^。Jurewicz等^[13]^的报道显示,尿中对羟基苯甲酸酯水平与精子形态、活力和血清睾酮水平下降显著相关。然而,其他几项研究结果并未发现酚类物质与精子质量的统计学关联^[14⇓-16]^。由于目前的研究结果尚不一致,并且大部分研究仍局限于酚类化合物单一物质暴露的影响,忽视了现实情况中多种酚类物质混合暴露的影响,酚类化合物混合暴露与男性精液质量的关联仍需进一步探讨。

然而,多种酚类化合物的同时分析具有挑战性,因为它们具有不同的极性范围和不同的理化性质。考虑到环境酚类在人体内广泛存在,迫切需要开发一种同时测定多种类别酚类化合物的方法。此外,酚类化合物在人体中的暴露处在痕量水平以及所测定生物基质的复杂性,都对分析方法的开发构成了重大挑战。尽管液相/气相色谱-串联质谱法(LC-MS/MS或GC-MS/MS)已被证明是用于痕量分析的强大工具^[17]^,但现有的检测方法在大规模流行病学研究中仍然存在不足。传统的预处理操作(如液液萃取(LLE)、固相萃取(SPE))耗时,并且需要大量的尿样和有害的有机溶剂^[18]^。为了节省样本、节约成本和时间,改进预处理过程非常重要。

基于此,课题组前期开发了一种可同时检测人体尿液中18种酚类化合物(包括对羟基苯甲酸酯、双酚类、羟基苯甲酮类和抗菌剂)的HPLC-MS/MS方法^[19]^,并开展了大样本流行病学研究对象的实际检测应用。本文以2021年12月-2022年11月上海市人类精子库捐献精子的志愿者为研究对象,分析酚类化合物单一暴露和混合暴露与男性精液质量参数间的关联。

## 1 实验部分

### 1.1 仪器与试剂

Agilent 1290-6490高效液相色谱-三重四极杆质谱仪(美国Agilent公司); 7080全自动生化分析仪(日本Hitachi公司); Nikon eclipse 50i显微镜(北京中仪光科科技发展有限公司); CPA225D电子分析天平(0.00001 g)(德国Satorious公司); Vortex-Genie2旋涡混合器(美国Scientific Industries公司); Thermo SPE121P离心浓缩系统和CL31R Multispeed离心机(美国Thermo公司)。

甲醇、乙腈、异丙醇、乙酸乙酯均为色谱纯,*β*-葡萄糖醛酸酶(85000 unit/mL),以上均购自德国Merck公司。

对羟基苯甲酸甲酯(MeP)、对羟基苯甲酸乙酯(EtP)、对羟基苯甲酸丙酯(PrP)、对羟基苯甲酸丁酯(BuP)、对羟基苯甲酸正庚酯(HeP)、对羟基苯甲酸苄酯(BzP)、BPA、双酚B(BPB)、双酚S(BPS)和三氯生(TCS)均购自德国Dr. Ehrenstorfer公司;双酚AF(BPAF)、双酚P(BPP)、双酚Z(BPZ)、2,4-二羟基二苯甲酮(BP-1)、2,2',4,4'-四羟基二苯甲酮(BP-2)、2,2'-二羟基-4-甲氧基二苯甲酮(BP-8)和4-羟基二苯甲酮(4-HBP)购自加拿大多伦多TRC公司;双酚AP(BPAP)购自美国New Haven AccuStandard公司。内标MeP-^13^C_6_购自加拿大多伦多TRC公司,内标BPA-d_16_购自加拿大CDN Isotopes公司,TCS-d_3_购自德国Dr. Ehrenstorfer公司。

每一类物质对应一种内标:双酚类,含BPA、BPS、BPB、BPP、BPAP、BPAF和BPZ,内标为BPA-d_16_;对羟基苯甲酸酯类,含MeP、EtP、PrP、BzP、BuP和HeP,内标为MeP-^13^C_6_;羟基苯甲酮类,含BP-1、BP-2、BP-8和4-HB,内标为BPA-d_16_;三氯生内标为TCS-d_3_。

### 1.2 标准溶液的配制

准确称取一定量的酚类标准品,溶于甲醇,配制成1.0 mg/mL的单标储备液。依次吸取18种酚类标准品储备液于甲醇中,涡旋混匀,配制成100.0 ng/mL混标母液,置于-20 ℃保存。吸取混标母液,以80%甲醇水溶液作溶剂,配制成10和100 ng/mL的2个混合标准应用液^[19]^。同时准确称取一定量的内标,溶于甲醇,配制成1.0 μg/mL的内标母液。依次吸取3种内标母液于甲醇中,涡旋混匀,配制成100.0 ng/mL内标工作液。取100 μL空白尿液,加入一定量上述混合标准应用液和内标工作液,经过样品前处理后,配制成质量浓度分别为0.2、0.5、1.0、5.0、10.0、20.0、30.0、40.0、50.0、60.0、80.0 ng/mL的系列标准溶液。

### 1.3 样品前处理

方法同前期报道^[19]^。取200 μL尿液,在其中加入20 μL内标工作液以及50 μL酶工作液(母液用水稀释100倍)混合。轻微涡旋10 s至混匀后,放置于37 ℃孵育过夜。孵育结束后,每管加入500 μL乙酸乙酯后涡旋10 s混匀。将混合液置于离心机中在4 ℃状态下15000 r/min离心10 min。管内溶液发生明显的分层,取出分界线上层的液体置于EP管中。在剩余的尿液层中继续加入500 μL乙酸乙酯后涡旋10 s混匀。将混合液置于离心机在4 ℃状态下15000 r/min离心10 min。管内溶液发生明显的分层,取出分界线上层的液体置于EP管中。两次萃取共同得到的液体为萃取液。将萃取液置于离心浓缩仪,于40 ℃浓缩至近干,加入200 μL 80%甲醇水溶液,涡旋10 s使之溶解充分。将混合液置于离心机中在4 ℃状态下15000 r/min离心3 min。最后将上清液转移到内插管色谱瓶中以备HPLC-MS/MS检测。

### 1.4 分析条件

色谱条件 Poroshell 120 EC-C18色谱柱(100 mm×3.0 mm, 2.7 μm; Agilent);柱温:40 ℃;流速:0.5 mL/min;进样量:10 μL。流动相:(A)纯水,(B)甲醇。梯度洗脱程序如下:0~5.0 min, 25%B~95%B; 5.0~11.0 min, 95%B; 11.0~11.1 min, 95%B~25%B; 11.1~13.0 min, 25%B。

质谱条件 电喷雾离子源(ESI),负离子模式,多重反应监测(MRM)模式扫描,离子源温度250 ℃,毛细管电离电压3000 V,雾化气压力137.895 kPa。其他质谱参数见文献[19]。

### 1.5 尿液样本采集

本研究以在上海市人类精子库初次捐精的志愿者为研究对象。自2021年12月起招募志愿者,研究对象的纳入标准如下:1)18~45周岁;2)上海常住户口;3)无遗传病史、性传播病史、慢性系统性疾病史如高血压、糖尿病等;4)禁欲时间≥2天。排除标准如下:1)有相关的环境暴露(例如油漆工人、胶厂工人等); 2)有影响精液质量的疾病史(例如疝修补并发睾丸萎缩、睾丸损伤、隐睾、输精管结扎、附睾炎、精囊炎、精索静脉曲张、泌尿系统疾病如膀胱炎、糖尿病以及其他内分泌疾病)。本研究研究对象均已签署项目知情同意书,本研究已通过上海交通大学附属新华医院伦理审查委员会批准(批件号:XHEC-C-2020-114-1)。

截至2022年11月,本研究共纳入1035例受试者。排除缺少酚类检测数据(*N*=183)、精液指标数据(*N*=31)和重要协变量(*N*=22)的样本后,本研究最终纳入799例样本进行分析。受试者尿液样本于入组当日采集,并置于-20 ℃冰箱冷冻直至检测。

### 1.6 尿样中酚类物质检测质量控制

使用儿童尿液作为空白基质用于方法的优化、验证和质量控制。儿童尿液采集于上海优生儿童队列随访期,将尿样(女生与男生的比例为3∶4)混合后进行检测,发现其中目标物浓度稳定且痕量(MeP 0.23 ng/mL, EtP 0.04 ng/mL, PrP 0.11 ng/mL, BPA 0.13 ng/mL,其他均<LOD),因此将其作为空白基质。

同时为了监测整个过程的本底,每间隔20个样品设置两个空白样(超纯水和儿童尿液)。为确保方法的稳定性,每间隔20个样品设置两个质量控制浓度点,包括低添加水平质量控制(QC_low_, 1 ng/mL)和高添加水平质量控制(QC_high_, 10 ng/mL)。

### 1.7 精液质量和尿液中肌酐浓度检测

精液标本通过手淫收集,采集前需禁欲3~7天。精液按照标准方案进行处理。精液分析按照世界卫生组织(WHO)《人类精液检验实验室手册》的建议进行。分析工作由人工完成。为减少精子特征评估中的差异,每次对每个精液样本分析两次。研究期间,进行了内部质量控制,以确保技术人员的结果之间没有明显差异。通过精液分析仪测量精液指标,包含精子浓度、精子总数、精液体积和精子前向运动百分比。精液量用刻度吸管进行评估。为了评估精子浓度和活力,将10 μL混合均匀的精液放入干净的标记室,用盖玻片轻轻盖住,然后在总放大倍率为×200的条件下进行检查。在显微镜视野中的100个方格中随机扫描10个,用细胞计数器记录精子数量。精子总数由精液量×精子浓度计算得到。在这项研究中,进行了内部质量控制,以确保精液参数之间没有显著差异。

尿肌酐浓度按照肌氨酸氧化酶法使用全自动生化分析仪检测,用于校正酚类浓度。

### 1.8 统计分析

本研究采用描述性统计学方法对研究人群基本资料和污染物分布特征进行分析。对符合正态分布的连续变量用平均值±标准差来表示,非正态分布的用中位数(四分位间距)来表示,分类资料采用数值(百分比)表示。样品检测结果中低于方法检出限的值以LOD/ $\sqrt{2}$ 代替,检出率低于80%的物质将不再纳入后续统计分析。由于酚类化合物浓度、精子浓度和精子总数呈偏态分布,我们采用自然对数转换后进行后续分析。

采用多元线性回归模型分析单污染物与精液质量间的关联,并通过文献检索纳入可能的混杂因素进一步调整。纳入的协变量为:年龄(连续型变量)、身体质量指数(BMI, 连续型变量)、教育水平(分类变量)、禁欲时间(分类变量)、吸烟(分类变量)、饮酒(分类变量)和尿液肌酐浓度(连续型变量)。此外,我们还进一步将污染物浓度分成四分位,分别是:Q1 (quantile 1,即P0~P25), Q2 (quantile 2,即P25~P50), Q3 (quantile 3,即P25~P75)和Q4 (quantile 4,即P75~P100)。以Q1作为对照,分析单污染物浓度与精液指标间的关联。

由于污染物间存在共线性,为了探索多污染物混合暴露与精液质量间的关系,本研究进一步采用加权分位数之和模型(weighted quantile sum, WQS)探索多污染物与精液质量间的关联。WQS模型是一种可以计算多种环境化合物累积混合效应的模型,并可以估计每种污染物的加权贡献值,有助于确定混合物中贡献最大的化合物^[20,21]^。该模型已经广泛应用于环境流行病学研究^[22⇓-24]^。由于并不确定酚类对精液质量影响的正负方向,我们分别对正负方向进行分析。

所有分析均使用R 4.1.1软件实现,R包“gWQS”用于实现WQS模型。

## 2 结果与讨论

### 2.1 人口学特征

本研究中799名研究对象基本人口学特征如表1所示,平均年龄和BMI分别为26.11岁和22.92 kg/m^2^,大部分研究对象报告从不吸烟(72.6%)和偶尔饮酒(64.8%)。共有517(64.7%)名研究对象有本科及以上学历。

**表1:** 研究对象的人口学特征

Characteristic	*n*		(%)
Abstinence time/d			
<3	178		(22.3)
3-8	590		(73.8)
≥8	31		(3.9)
Smoking status			
Never	580		(72.6)
Former	105		(13.1)
Current	114		(14.3)
Alcohol consumption			
Never	219		(27.4)
Occasionally	518		(64.8)
Usually	62		(7.8)
Educational level			
Below bachelor	282		(35.3)
At bachelor	353		(44.2)
Above bachelor	164		(20.5)
Age/years (Mean±SD)^*^		26.11±5.70	
BMI/(kg/m^2^) (Mean±SD)^*^		22.92±2.77	
Creatinine/(μmol/L) (Mean±SD)^*^		12187±7394	

*n* (%): number of volunteers and the corresponding percentage; BMI: body mass index. * *n*=799.

### 2.2 尿液中酚类物质的浓度分布

研究对象尿液中酚类物质的浓度分布见表2。其中,除BPAF、BPZ、BzP、BuP、HeP、BP-1、BP-2、BP-8、4-HBP外,其他9种物质的检出率均大于80%,将这9种化合物进一步纳入后续数据分析。

**表2:** 尿液中酚类化合物的质量浓度分布及检出率

Compound	LOD/ (ng/mL)	Mass concentrations of phenols/(ng/mL)							Detection rate/%
		Min	Max	P10	P25	Median	P75	P90	
Bisphenols (BP)									
BPA	0.08	<LOD	43.22	0.01	0.20	0.56	1.63	4.08	85.73
BPS	0.003	<LOD	270.05	0.02	0.05	0.23	0.70	1.44	94.99
BPB	0.0003	<LOD	0.90	<LOD	0.003	0.01	0.03	0.08	84.98
BPP	0.0001	<LOD	15.21	<LOD	0.004	0.02	0.08	0.27	81.10
BPAP	0.002	<LOD	4.59	<LOD	0.004	0.02	0.06	0.17	81.98
BPAF	0.001	<LOD	0.66	<LOD	<LOD	<LOD	0.02	0.05	35.79
BPZ	0.01	<LOD	0.60	<LOD	<LOD	<LOD	0.03	0.06	48.69
Parabens									
MeP	0.002	0.033	599.87	1.58	3.23	8.73	28.53	72.76	100
EtP	0.005	<LOD	1603.10	<LOD	1.40	2.49	5.47	15.41	84.61
PrP	0.0005	<LOD	568.01	0.15	0.71	2.65	8.84	26.28	93.37
BzP	0.003	<LOD	0.22	<LOD	<LOD	<LOD	<LOD	0.005	16.02
BuP	0.002	<LOD	68.95	<LOD	<LOD	<LOD	<LOD	0.01	23.53
HeP	0.002	<LOD	0.18	<LOD	<LOD	<LOD	<LOD	<LOD	9.89
Benzophenones									
BP-1	0.005	<LOD	131.99	<LOD	0.01	0.02	0.08	0.28	78.35
BP-2	0.002	<LOD	25.01	<LOD	<LOD	<LOD	0.01	0.02	36.92
BP-8	0.05	<LOD	25.02	<LOD	<LOD	<LOD	0.07	0.54	29.54
4-HBP	0.01	<LOD	3.04	<LOD	0.01	0.04	0.09	0.22	75.47
Antimicrobial									
TCS	0.002	<LOD	342.89	0.01	0.09	0.31	1.09	6.44	91.24

P10: 10th percentile; P25: 25th percentile; P75: 75th percentile; P90: 90th percentile. MeP: methyl 4-hydroxybenzoate; EtP: ethyl 4-hydroxybenzoate; PrP: propyl 4-hydroxybenzoate; BzP: benzyl 4-hydroxybenzoate; BuP: butyl 4-hydroxybenzoate; HeP: heptyl 4-hydroxybenzoate; BP-1: 2,4-dihydroxy benzophenone; BP-2: 2,2',4,4'-tetrahydroxy benzophenone; BP-8: 2,2'-dihydroxy-4-methoxy benzophenone; 4-HBP: 4-hydroxy benzophenone; TCS: triclosan.

### 2.3 尿液中单一酚类化合物浓度与精液质量的关联分析

如表3所示,未调整和调整后的多元线性模型结果提示,尿液中EtP浓度与精子浓度、精子总数和精液体积呈显著负相关(*P*<0.05)。如图1所示,当将酚类浓度按四分位分类后,与Q1组相比,EtP浓度Q4组和MeP浓度Q3组的男性精子浓度显著降低(*P*<0.05)。此外,与Q1相比,EtP浓度Q2、Q3和Q4组均与精子总数呈现显著的负相关。本研究表明,对羟基苯甲酸酯类化合物可能与男性精液质量下降显著相关,这与一项既往研究^[25]^结果类似:生育与环境纵向调查(The Longitudinal Investigation of Fertility and the Environment, LIFE)招募了501对夫妇,分析其丈夫尿液中对羟基苯甲酸酯类浓度与精液质量的关系,结果显示,EtP与精子数量、精子活力之间存在反向关系,MeP与精子活力低之间存在显著关联。然而,另外几项人群研究并未发现对羟基苯甲酸酯类化合物与精液质量间的显著关联^[26,27]^。本研究并未发现双酚类化合物和三氯生与男性精液质量的关联。该结果与几项同样在普通人群中开展的研究相似^[14⇓-16]^。然而,Meeker等^[12]^的一项横断面研究测量了190名寻求不育治疗的男性尿液中BPA浓度,其研究结果提示BPA浓度与精子浓度、形态异常和精子活力呈负相关。Vitku等^[28]^的研究结果与其一致。Vitku等在不育男性中测量其血浆和精液中BPA浓度,其研究表明随着男性不育程度的提高,血浆中BPA水平增加,与健康男性相比,轻度和中度不育症男性血浆BPA水平更高,同时BPA浓度也与精子数量、精子形态呈负相关。Lassen等^[29]^的研究结果仅发现了尿液中BPA与精子活力呈负相关。造成这种不一致结果的原因可能是由于人群特征、样本量和酚类物质暴露水平的差异。

**表3:** 尿液中酚类物质含量与精液质量参数的多元线性回归结果

Compound	*β* (95% CI)^a)^										
	Semen concentration^b)^			Total sperm count^b)^			Semen volume			Progressive motility	
	Crude	Adjusted		Crude	Adjusted		Crude	Adjusted		Crude	Adjusted
Bisphenol											
BPA	-0.003 (-0.037, 0.031)	0.002 (-0.035, 0.039)		-0.019 (-0.064, 0.026)	0.004 (-0.041, 0.049)		-0.018 (-0.090, 0.054)	0.023 (-0.054, 0.100)		0.188 (-0.551, 0.927)	-0.025 (-0.827, 0.777)
BPS	-0.020 (-0.046, 0.006)	-0.019 (-0.047, 0.009)		-0.033 (-0.068, 0.002)	-0.031 (-0.066, 0.004)		-0.031 (-0.087, 0.025)	-0.029 (-0.087, 0.029)		0.368 (-0.210, 0.946)	0.279 (-0.325, 0.883)
BPB	-0.014 (-0.039, 0.011)	-0.010 (-0.038, 0.018)		-0.031 (-0.064, 0.002)	-0.007 (-0.040, 0.026)		-0.012 (-0.065, 0.041)	0.039 (-0.019, 0.097)		-0.070 (-0.614, 0.474)	-0.212 (-0.812, 0.388)
BPP	-0.004 (-0.021, 0.013)	-0.001 (-0.020, 0.018)		-0.015 (-0.038, 0.008)	-0.001 (-0.024, 0.022)		-0.020 (-0.057, 0.017)	0.004 (-0.036, 0.044)		0.068 (-0.311, 0.447)	-0.004 (-0.418, 0.410)
BPAP	-0.004 (-0.032, 0.024)	0.000 (-0.030, 0.030)		-0.015 (-0.052, 0.022)	-0.002 (-0.039, 0.035)		-0.018 (-0.077, 0.041)	0.003 (-0.060, 0.066)		-0.177 (-0.791, 0.437)	-0.394 (-1.047, 0.259)
Paraben											
MeP	-0.029 (-0.062, 0.004)	-0.028 (-0.063, 0.007)		-0.024 (-0.068, 0.020)	-0.010 (-0.054, 0.034)		0.015 (-0.056, 0.086)	0.043 (-0.030, 0.116)		0.241 (-0.487, 0.969)	0.240 (-0.514, 0.994)
EtP	-0.022 (-0.040, -0.004)	-0.023 (-0.041, -0.005)		-0.039 (-0.062, -0.016)	-0.036 (-0.059, -0.013)		-0.052 (-0.089, -0.015)	-0.042 (-0.080, -0.004)		0.050 (-0.338, 0.438)	-0.015 (-0.407, 0.377)
PrP	-0.014 (-0.032, 0.004)	-0.013 (-0.031, 0.005)		-0.014 (-0.037, 0.009)	-0.006 (-0.029, 0.017)		0.003 (-0.034, 0.040)	0.020 (-0.018, 0.058)		-0.150 (-0.536, 0.236)	-0.154 (-0.551, 0.243)
TCS	0.011 (-0.009, 0.031)	0.010 (-0.011, 0.031)		0.015 (-0.012, 0.042)	0.012 (-0.015, 0.039)		-0.007 (-0.050, 0.036)	-0.008 (-0.051, 0.035)		0.165 (-0.278, 0.608)	0.141 (-0.307, 0.589)

a) Values were adjusted for age (continuous), BMI (continuous), abstinence period (categorical), smoking status (categorical), alcohol consumption (categorical), educational level (categorical), and creatinine level (continuous). b) Values were transformed using the natural logarithm. *β*: regression coefficient; CI: confidence interval. Values in bold indicate statistical significance (*P*<0.05).

**图1:**
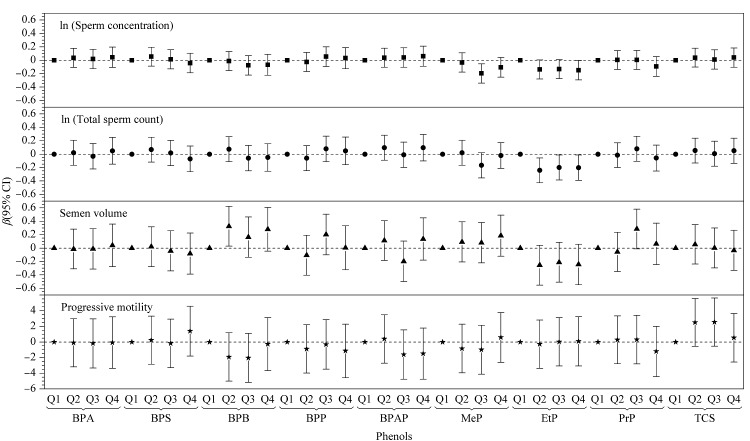
尿液中酚类浓度(四分位)与精液质量参数的多元线性回归结果

对羟基苯甲酸酯与精液质量之间的负向关联可能与酚类物质的内分泌干扰特性相关。研究表明,对羟基苯甲酸酯类化合物可作为内分泌干扰物,具有与内源性雌激素(17*β*-雌二醇)或抗雄激素类似的活性,导致人体内促性腺激素和睾丸睾酮水平发生变化^[30]^。因此,它们可能会干扰正常的精子生成过程。另一项研究^[31]^提示了对羟基苯甲酸酯导致精液质量下降的另一可能潜在机制。该研究对三周龄雄性Sprague-Dawley大鼠(*n*=8)采取对羟基苯甲酸丁酯灌胃染毒(剂量为1000 mg/kg)进行研究,发现对羟基苯甲酸丁酯可能通过破坏Sertoli细胞的波形蛋白丝导致精细胞过早脱离Sertoli细胞。由于失去了Sertoli细胞提供的营养支持,过早释放的精细胞可能会发生凋亡。此外,对羟基苯甲酸酯类化合物还可能通过诱导过量活性氧产生,破坏精子发生^[32]^。

### 2.4 酚类混合暴露

本研究采用WQS模型进一步探索酚类化合物的混合暴露男性生殖健康风险,结果如图2和表4所示,WQS指数与精子浓度呈显著的负相关(*P*<0.05)。在调整了尿肌酐和潜在的混杂因素后,WQS指数每增加1个单位,精子浓度的对数值减少0.103个单位。其中MeP和EtP是影响精子浓度权重最高的化合物。既往探索酚类化合物混合暴露对男性生殖系统毒性的相关研究十分有限。一项体外研究探讨了对羟基苯甲酸酯类混合物(MeP、EtP、PrP、BuP和iso-BuP)的雄激素受体(AR)拮抗作用。该研究结果表明,对羟基苯甲酸酯混合物对AR受体的拮抗作用高于单种化合物作用,提示混合物毒性会产生一定的相加效应^[33]^。这个体外研究的结果与我们在人类流行病学研究中的发现相类似,WQS模型得出的估计值高于单一污染物分析得出的估计值。由于人类处于多种污染物混合暴露中,混合暴露在人群中的健康影响不容忽视,亟需进一步深入研究。

**图2:**
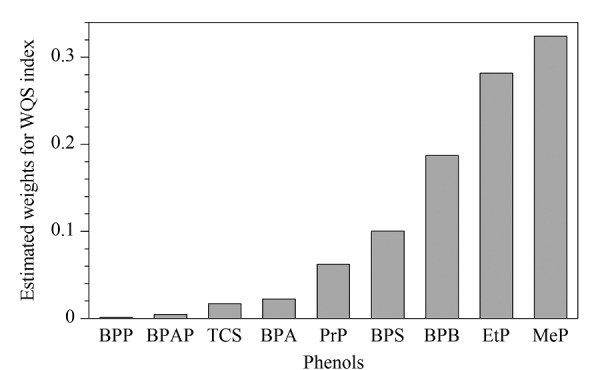
与精子浓度相关的WQS指数中各酚类的权重

**表4:** WQS指数与精液质量参数的WQS回归模型结果

Parameter	Positive			Negative	
	*β* (95% CI)^a)^	*P*-value		*β* (95% CI)^a)^	*P*-value
Semen concentration^b)^	0.027 (-0.045, 0.099)	0.470		-0.103 (-0.181, -0.025)	0.010
Total sperm count^b)^	0.027 (-0.075, 0.129)	0.605		-0.103 (-0.207, 0.001)	0.053
Semen volume	0.126 (-0.038, 0.290)	0.132		-0.096 (-0.247, 0.055)	0.214
Progressive motility	0.439 (-1.448, 2.326)	0.648		-0.906 (-2.640, 0.828)	0.306

a) Values were adjusted for age (continuous), BMI (continuous), abstinence period (categorical), smoking status (categorical), alcohol consumption (categorical), educational level (categorical), and creatinine level (continuous). b) Values were transformed using the natural logarithm. Values in bold indicate statistical significance (*P*<0.05).

相较于先前的研究,本研究具有以下几点优势。首先,本研究具有较大的样本量,确保了研究的统计效能。其次,我们的研究对象来自捐精志愿者,他们对自己的精液质量和环境酚类暴露情况并不了解,可以有效地避免选择偏倚。此外,我们的研究揭示了酚类化合物混合暴露与男性精液质量的潜在关联。

然而,本研究尚有一些局限性。首先,本研究是一项横断面研究,因此很难对暴露和结果进行因果推断。其次,酚类物质的浓度是通过单点尿液样本来测量的。由于酚类物质半衰期短,从体内消除速度较快,因此可能存在暴露错分的偏倚。同样地,单次精液质量结果也可能存在相同的偏倚。然而,Mahalingaiah等^[34]^的研究表明,单一尿液样本可用于预测BPA的长期(数周至数月)暴露。既往的研究^[35]^也表明,在流行病学研究中,一份精液样本足以评估研究对象的精液质量。此外,尿液中酚类浓度并不能直接代表男性生殖器官的暴露状态,未来仍需更多研究进行进一步探索。

## 3 结论

本研究发现,育龄男性普遍暴露于酚类物质中。酚类暴露可能与男性精液质量下降相关,主要表现在降低精子浓度和精子总数。后续应进一步开展大样本的前瞻性研究,探索酚类化合物暴露对男性生殖健康的影响。

## References

[1] Agarwal A, Baskaran S, Parekh N, et al. Lancet (London, England), 2021, 397(10271): 319 33308486 10.1016/S0140-6736(20)32667-2

[2] Salas-Huetos A, Bulló M, Salas-Salvadó J. Hum Reprod Update, 2017, 23(4): 371 28333357 10.1093/humupd/dmx006

[3] Greenberg D R, Bhambhvani H P, Basran S S, et al. J Urology, 2022, 208(2): 406 10.1097/JU.0000000000002682 PMC9283262 35344413

[4] Ou Z, Wen Q, Deng Y, et al. Reprod Biol Endocrinol, 2020, 18(1): 55 32460768 10.1186/s12958-020-00615-x PMC7251897

[5] Sharma R, Harlev A, Agarwal A, et al. Eur Urol, 2016, 70(4): 635 27113031

[6] Jeng H A, Pan C H, Lin W Y, et al. J Hazard Mater, 2013, 244/245: 436 23314003 10.1016/j.jhazmat.2012.11.008

[7] Zhou Y, Meng T, Wu L, et al. Environ Int, 2020, 135: 105364 31841801 10.1016/j.envint.2019.105364

[8] Kataria N, Bhushan D, Gupta R, et al. Environ Pollut, 2022: 120319 36183872 10.1016/j.envpol.2022.120319

[9] Zhao H, Zheng Y, Zhu L, et al. Environ Sci Technol, 2020, 54(6): 3447 32101413 10.1021/acs.est.9b07634

[10] Błedzka D, Gromadzińska J, Wᶏsowicz W. Environ Int, 2014, 67: 27 24657492 10.1016/j.envint.2014.02.007

[11] Xiao C, Wang L, Zhou Q, et al. J Hazard Mater, 2020, 384: 121488 31699483 10.1016/j.jhazmat.2019.121488

[12] Meeker J D, Calafat A M, Hauser R. Environ Sci Technol, 2010, 44(4): 1458 20030380 10.1021/es9028292 PMC2823133

[13] Jurewicz J, Radwan M, Wielgomas B, et al. J Occup Environ Med, 2017, 59(11): 1034 28692609 10.1097/JOM.0000000000001106

[14] Adoamnei E, Mendiola J, Vela-Soria F, et al. Environ Res, 2018, 161: 122 29156341 10.1016/j.envres.2017.11.002

[15] Mantzouki C, Bliatka D, Iliadou P K, et al. Food Chem Toxicol, 2019, 125: 562 30738989 10.1016/j.fct.2019.02.016

[16] Pollard S H, Cox K J, Blackburn B E, et al. Reprod Toxicol, 2019, 90: 82 31445078 10.1016/j.reprotox.2019.08.014 PMC6885548

[17] Vila M, Lamas J P, Garcia-Jares C, et al. J Chromatogr A, 2015, 1405: 12 26091782 10.1016/j.chroma.2015.05.061

[18] Grześkowiak T, Czarczyńska-Goślińska B, Zgoła-Grześkowiak A. TrAC-Trends Anal Chem, 2016, 75: 209

[19] Ao J, Zhang Q, Tang W, et al. Chemosphere, 2021, 278: 130494 33845434 10.1016/j.chemosphere.2021.130494

[20] Carrico C, Gennings C, Wheeler D C, et al. J Agr Biol Envir St, 2015, 20(1): 100 10.1007/s13253-014-0180-3 PMC6261506 30505142

[21] Czarnota J, Gennings C, Wheeler D C. Cancer Inform, 2015, 14(s2): 159 26005323 10.4137/CIN.S17295 PMC4431483

[22] Day D B, Collett B R, Barrett E S, et al. Environ Int, 2021, 147: 106330 33418196 10.1016/j.envint.2020.106330 PMC9291724

[23] Sun Y, Li X, Benmarhnia T, et al. Environ Int, 2022, 158: 106888 34563749 10.1016/j.envint.2021.106888 PMC9022440

[24] Wallace E R, Buth E, Szpiro A A, et al. Environ Res, 2023, 216: 114759 36370819 10.1016/j.envres.2022.114759 PMC9817935

[25] Smarr M M, Honda M, Kannan K, et al. Reprod Toxicol, 2018, 77: 103 29474822 10.1016/j.reprotox.2018.02.008 PMC5878147

[26] Adoamnei E, Mendiola J, Moñino-García M, et al. Sci Total Environ, 2018, 621: 201 29179076 10.1016/j.scitotenv.2017.11.256

[27] Nishihama Y, Toshima H, Yoshinaga J, et al. Environ Health Prev Med, 2017, 22(1): 5 29165110 10.1186/s12199-017-0618-7 PMC5661912

[28] Vitku J, Sosvorova L, Chlupacova T, et al. Physiol Res, 2015, 64(Suppl 2): S303 26680493 10.33549/physiolres.933090

[29] Lassen T H, Frederiksen H, Jensen T K, et al. Environ Health Perspect, 2014, 122(5): 478 24786630 10.1289/ehp.1307309 PMC4014766

[30] Liang J, Liu Q S, Ren Z, et al. Sci Total Environ, 2023, 869: 161793 36702264 10.1016/j.scitotenv.2023.161793

[31] Alam M S, Kurohmaru M. Acta Histochem, 2014, 116(5): 682 24444665 10.1016/j.acthis.2013.12.006

[32] Glander H G, Rytter M, Schönborn C. Zentralbl Gynakol, 1984, 106(9): 573 6741334

[33] Kjærstad M B, Taxvig C, Andersen H R, et al. Int J Androl, 2010, 33(2): 425 20132345 10.1111/j.1365-2605.2009.01034.x

[34] Mahalingaiah S, Meeker J D, Pearson K R, et al. Environ Health Perspect, 2008, 116(2): 173 18288314 10.1289/ehp.10605 PMC2235217

[35] Chiu Y H, Edifor R, Rosner B A, et al. Am J Epidemiol, 2017, 186(8): 918 28541378 10.1093/aje/kwx169 PMC5860180

